# Myriad Presentations of Intracranial Meningiomas: Pictoral Essay

**DOI:** 10.5334/jbsr.2751

**Published:** 2022-04-28

**Authors:** Peeyush Kumar Dhagat

**Affiliations:** 1 Armed Forces Medical College, Pune, IN

**Keywords:** Intracranial meningiomas, MRI

## Abstract

Meningiomas are the most common non-glial tumor of the central nervous system (CNS). Seen in middle age with a female preponderance, most of the tumors are solitary and supratentorial with benign histology (WHO grade I). Atypical and anaplastic (malignant) meningiomas (WHO grade II and III), comprise 15–20% of all intracranial meningiomas [1,2,3,4,5]. Magnetic resonance imaging (MRI) is the imaging modality of choice.

## Introduction

Meningiomas can involve the entire neuroaxis. Exposure to ionizing radiation is the only established risk factor [1,2].

**Figure 1:**
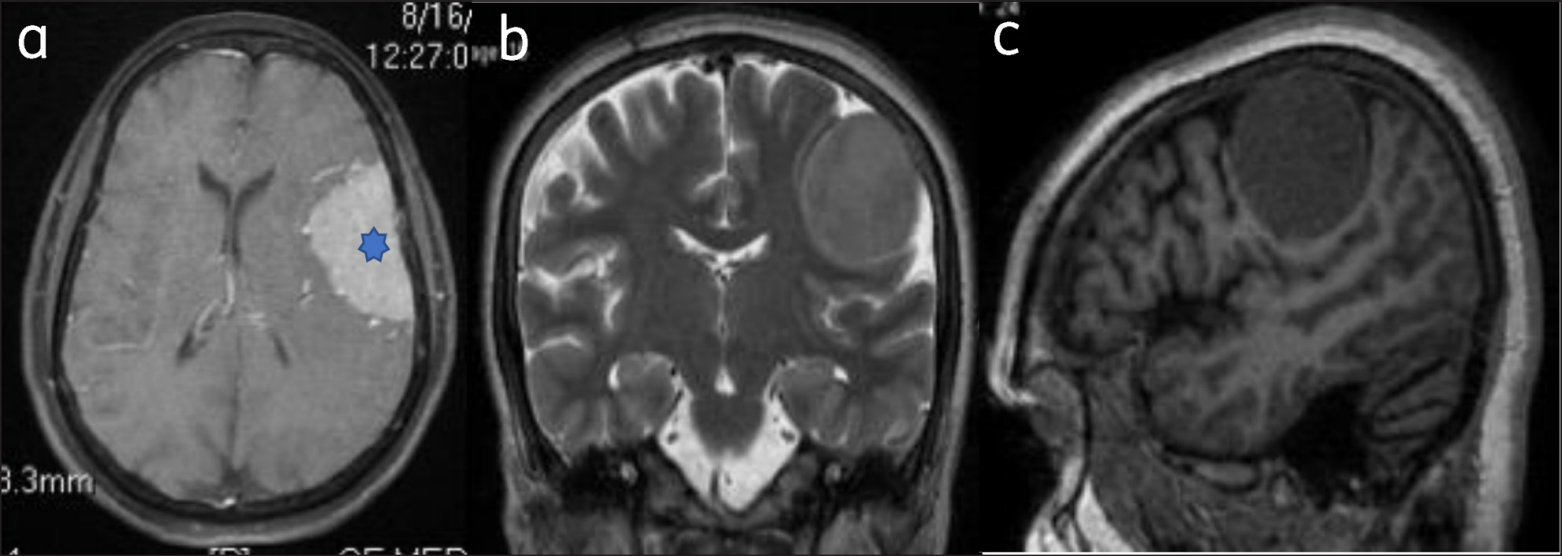
**Classical meningioma: –** (**a**) Axial post contrast image show enhancing broad based extra-axial lesion (star). Lesion is isointense on T2W (**b**) and T1W (**c**) to the gray matter.

Classical Meningioma are extra axial and broad based, appear hypo-isointense on T1WI and iso-hyperintense on T2WI to the gray matter, and show intense homogenous contrast enhancement (***Figure 1***).

**Figure 2:**
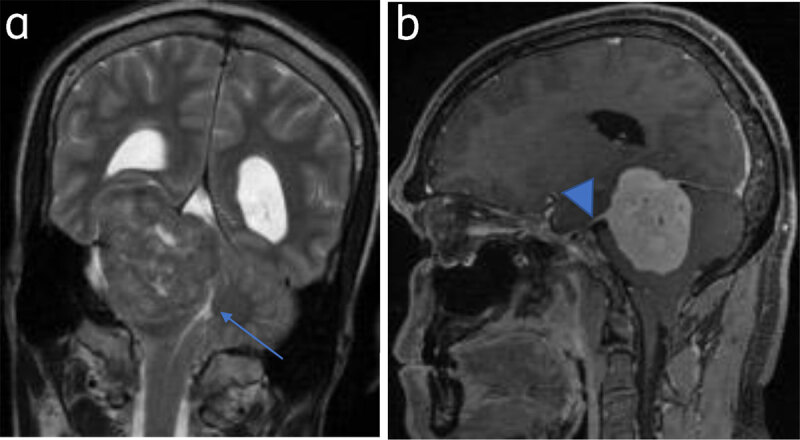
**Tentorial meningioma with dural tail:** (**a**) Coronal T2WI tentorial meningioma. Fourth ventricle is effaced (arrow). (**b**) Sagittal post contrast image shows dural tail (arrowhead).

“Dural tail sign” refers to focal (linear or patchy) dural enhancement adjacent to the tumor and can be extensive. Can also be seen in metastases, lymphomas, and some glial tumors [4] (***Figure 2***).

**Figure 3:**
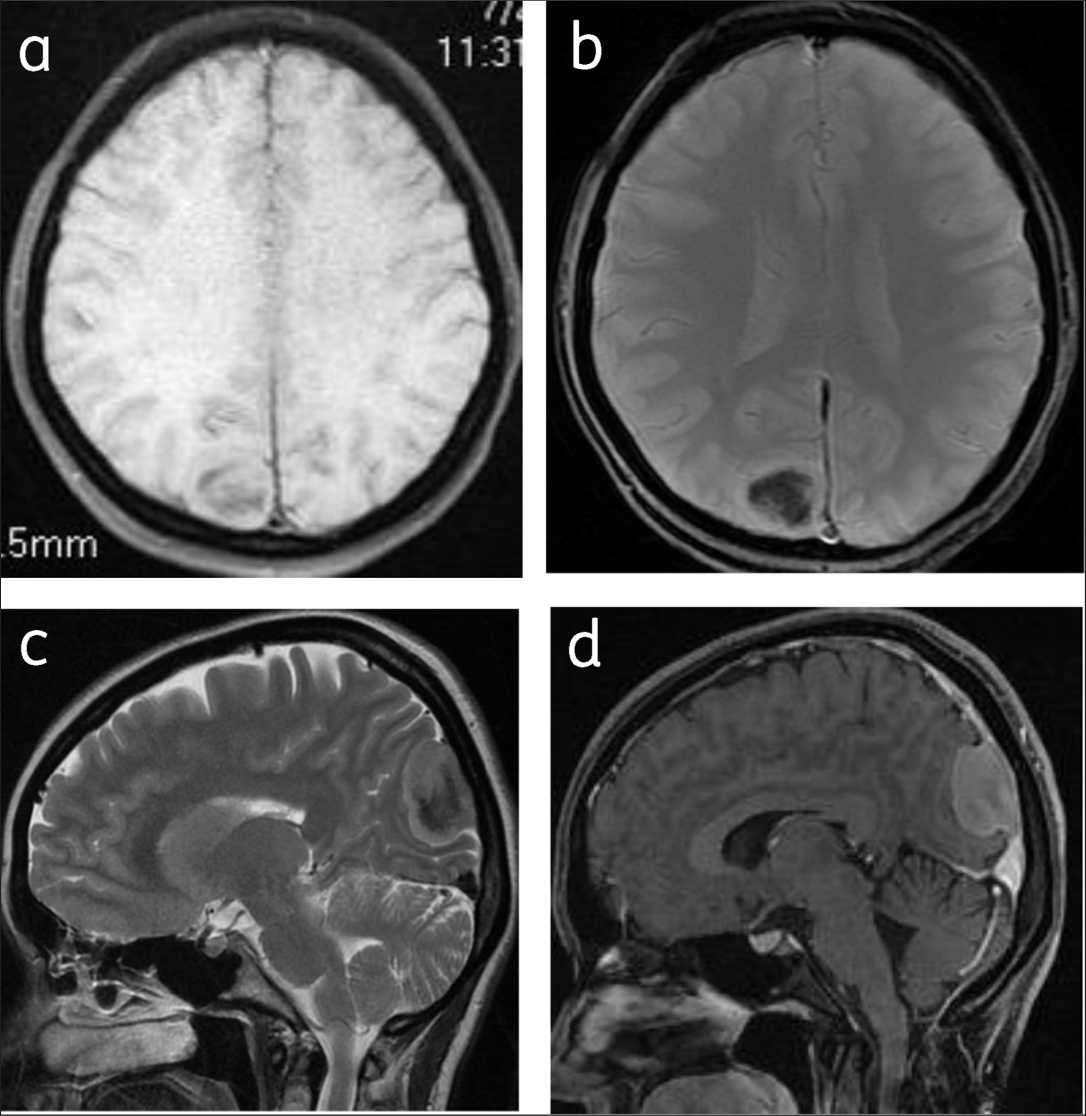
**Calcified meningioma:** Axial T1WI (**a**), T2* gradient (**b**), sagittal T2W (**c**) and sagittal post contrast (**d**) images shows right parafalcine calcified meningioma with hypointense central signal.

Approximately 25% show calcification [1] and appear hypointense on T1 and T2WI, with blooming on GRE (***Figure 3***).

**Figure 4:**
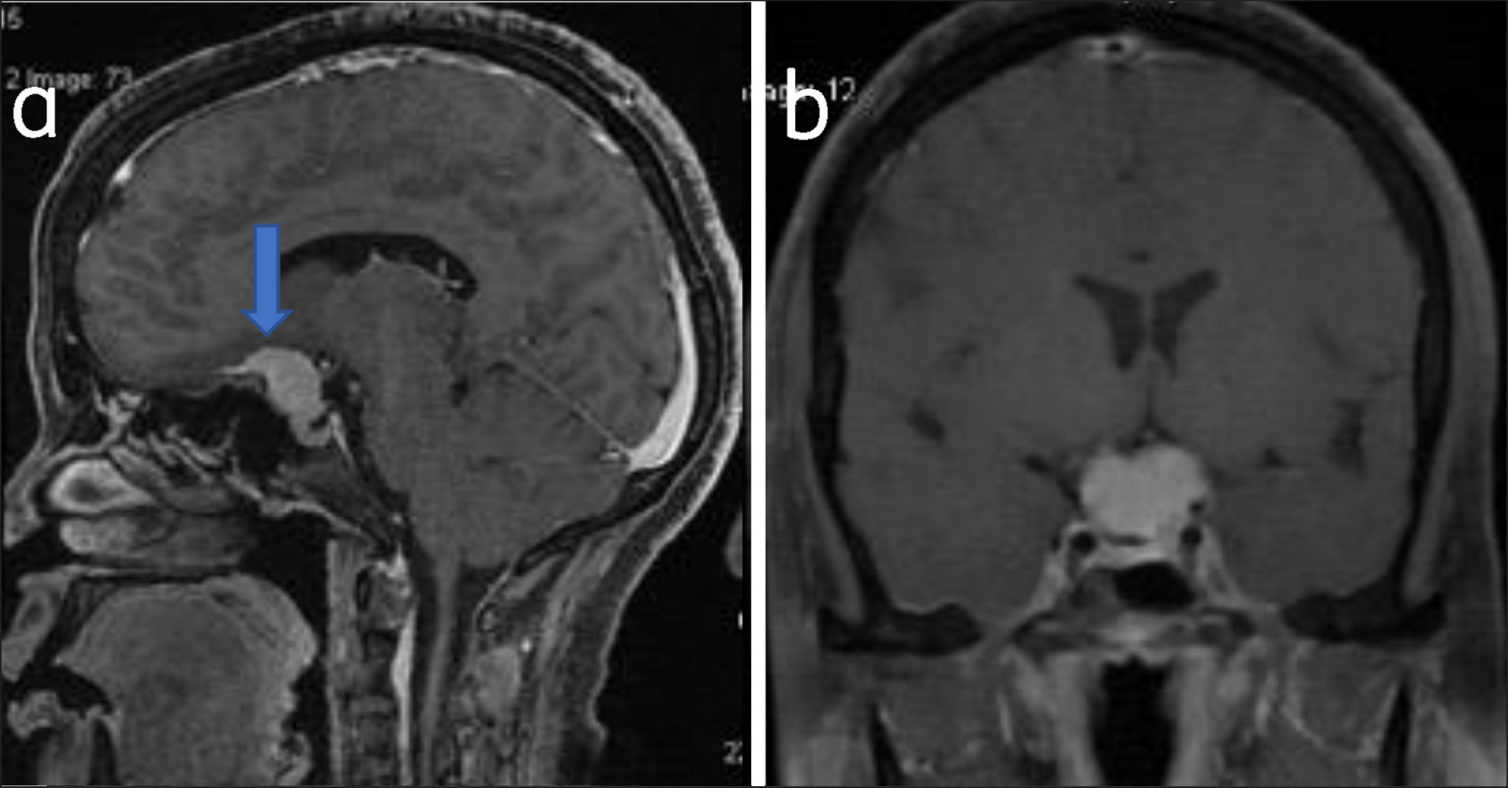
**Suprasellar meningioma:** Sagittal (**a**) and coronal (**b**) post-contrast T1W shows suprasellar meningioma (arrow).

**Figure 5:**
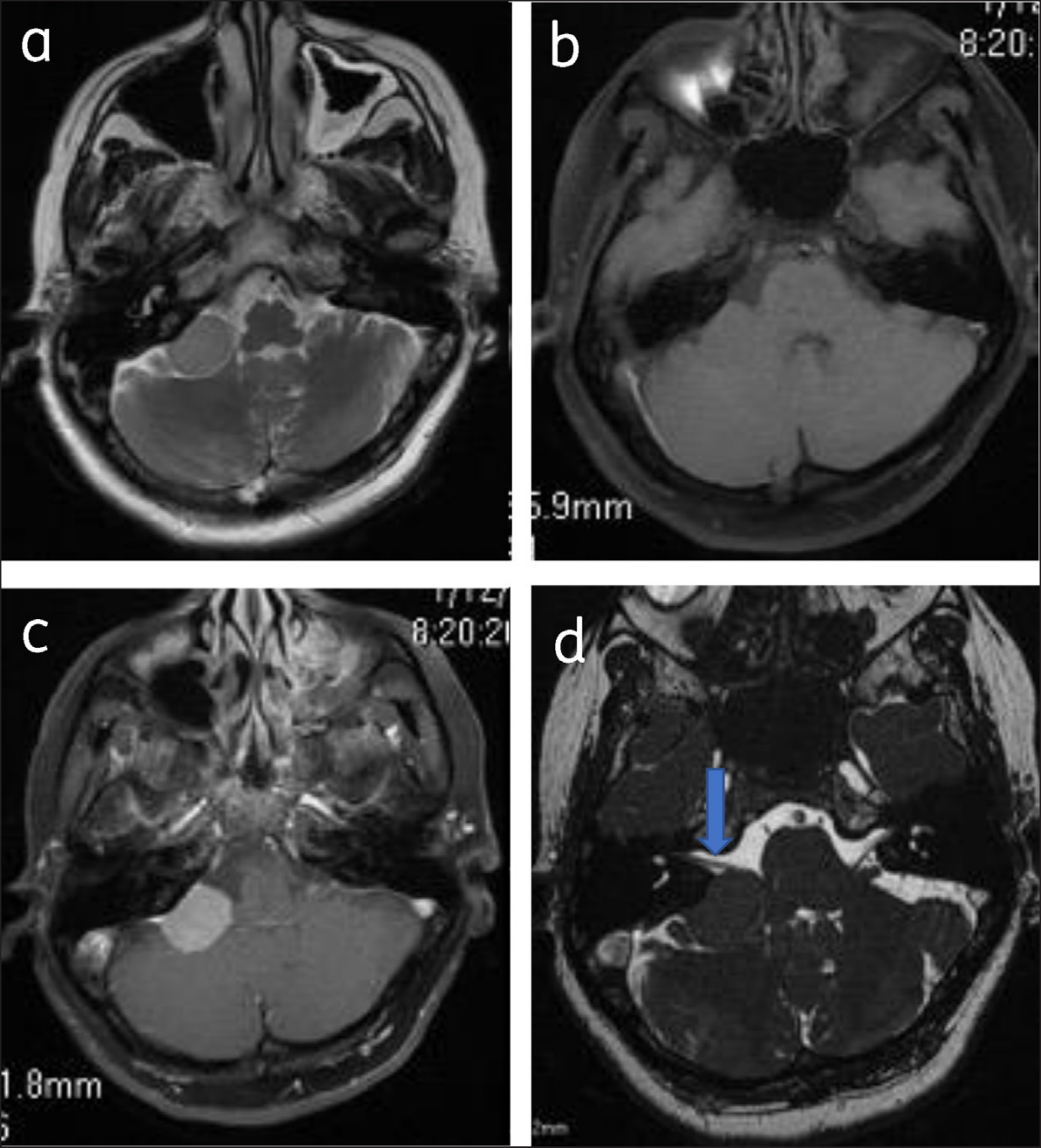
**Cerebellopontine angle meningioma:** Axial T2 (**a**), pre-contrast (**b**) and post contrast (**c**), show right CPA meningioma with dural tail. Axial 3DFIESTA (**d**) shows meningioma is indenting right VII–VIII^th^ nerve complex (arrow).

**Figure 6:**
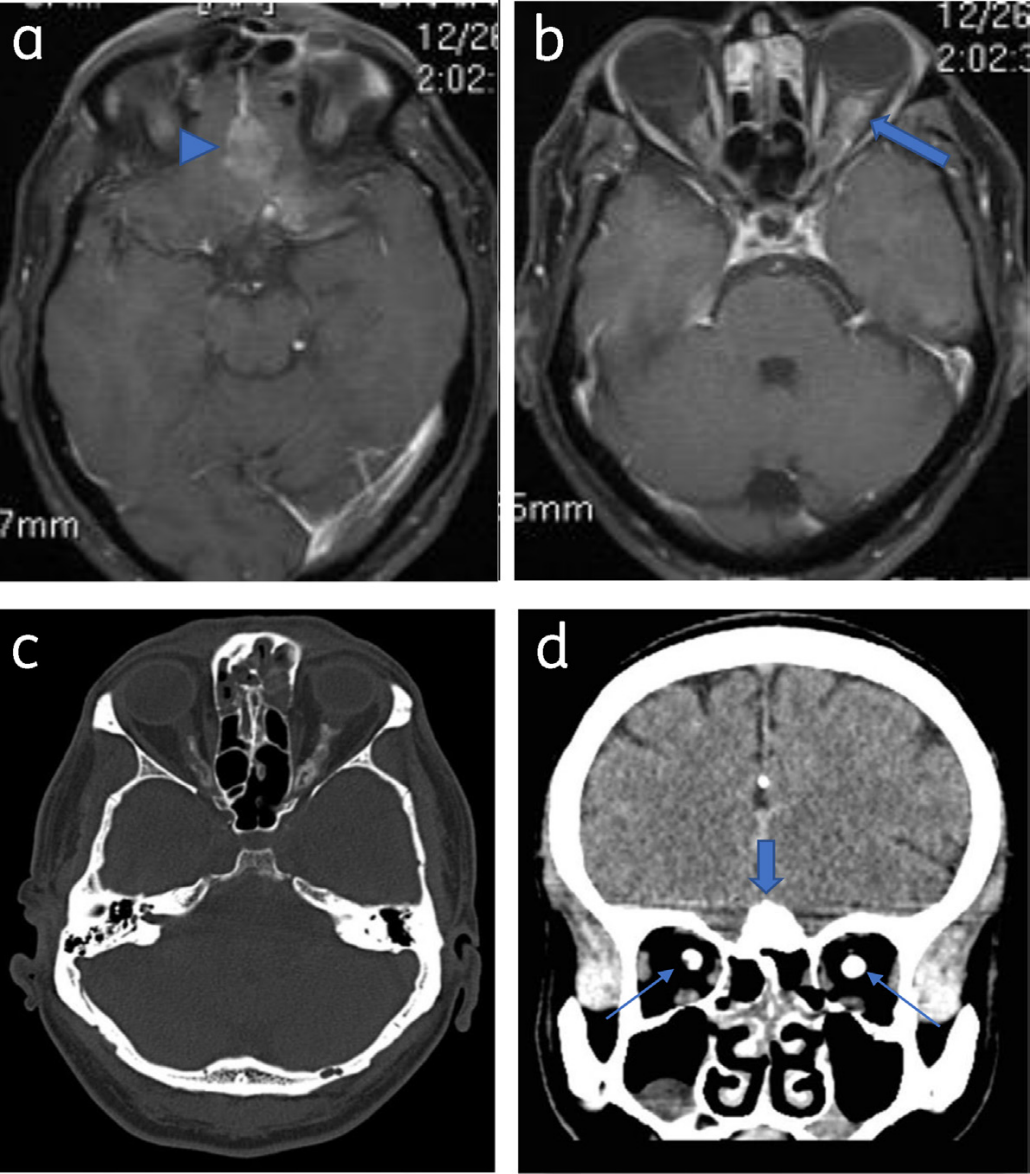
**Optic nerve sheath meningioma and olfactory meningioma:** Axial post contrast T1WI (**a**) and (**b**) shows olfactory grove meningioma (arrowhead) and bilateral optic nerve sheath meningioma (arrow). Axial (**c**) and coronal NCCT (**d**) shows calcification in the olfactory groove lesion (thick arrow) and both optic nerves (thin arrows).

**Figure 7:**
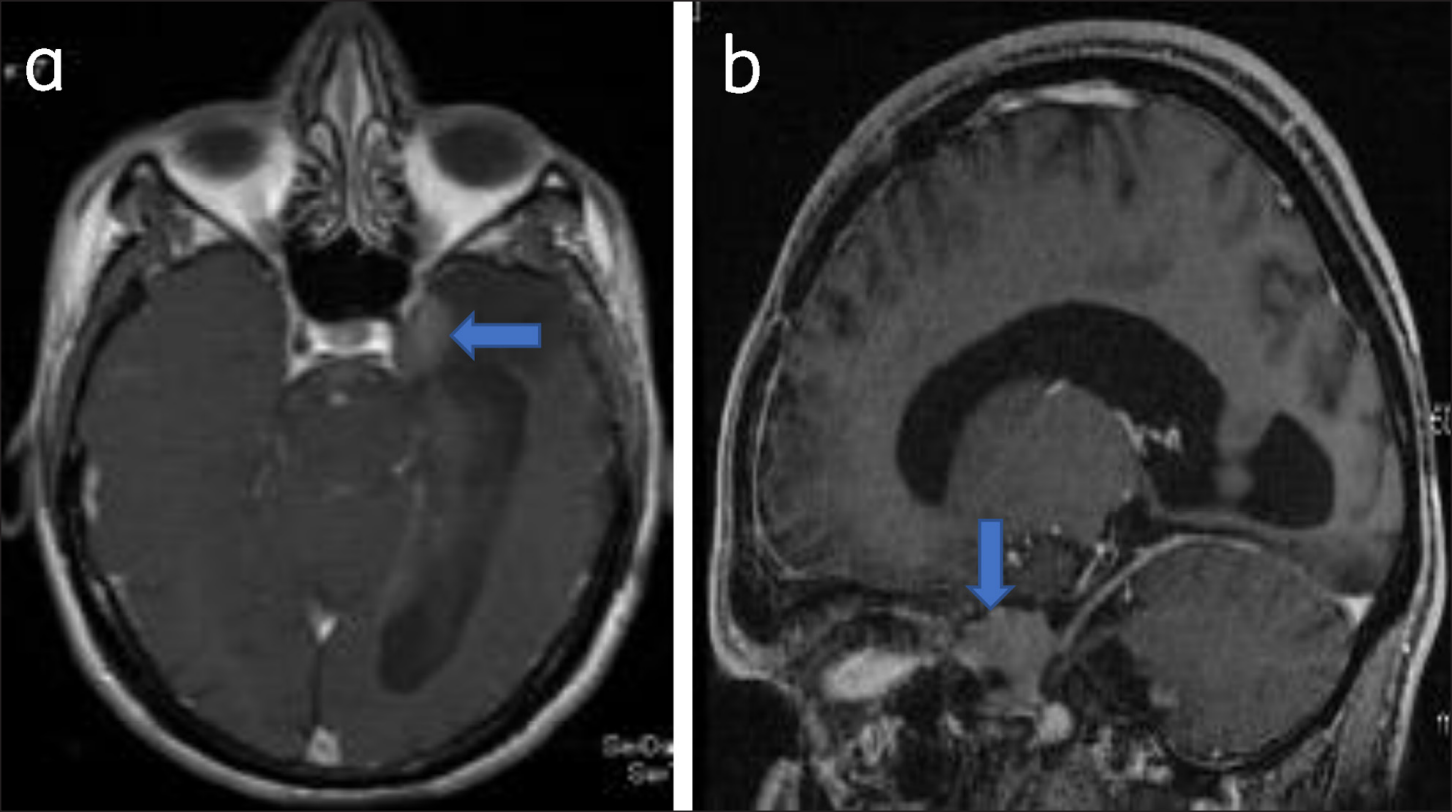
**Parasellar meningioma:** Axial (**a**) and sagittal (**b**) post-contrast T1W show a enhancing left parasellar meningioma (arrow).

**Figure 8:**
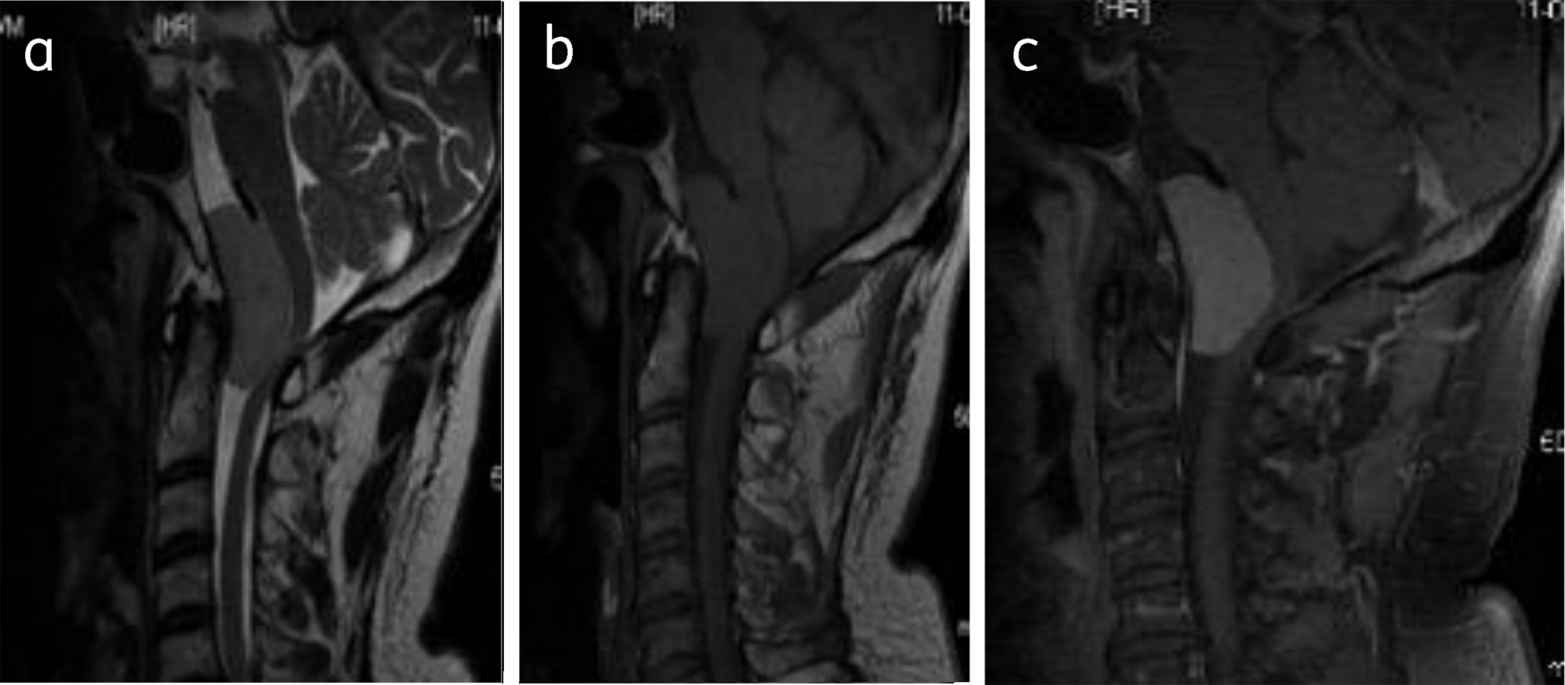
**Clival meningioma:** Sagittal T2WI (**a**), T1WI (**b**), and post contrast T1W (**c**) shows foramen magnum meningioma (asterisk) causing compressive myelopathy.

Common locations include parasagittal, convexity, and sphenoid wing. Uncommon locations include olfactory groove, optic nerve sheath, intraventricular, tentorial apex, and intraosseous and posterior fossa (***Figures 4, 5, 6, 7, 8***).

**Figure 9:**
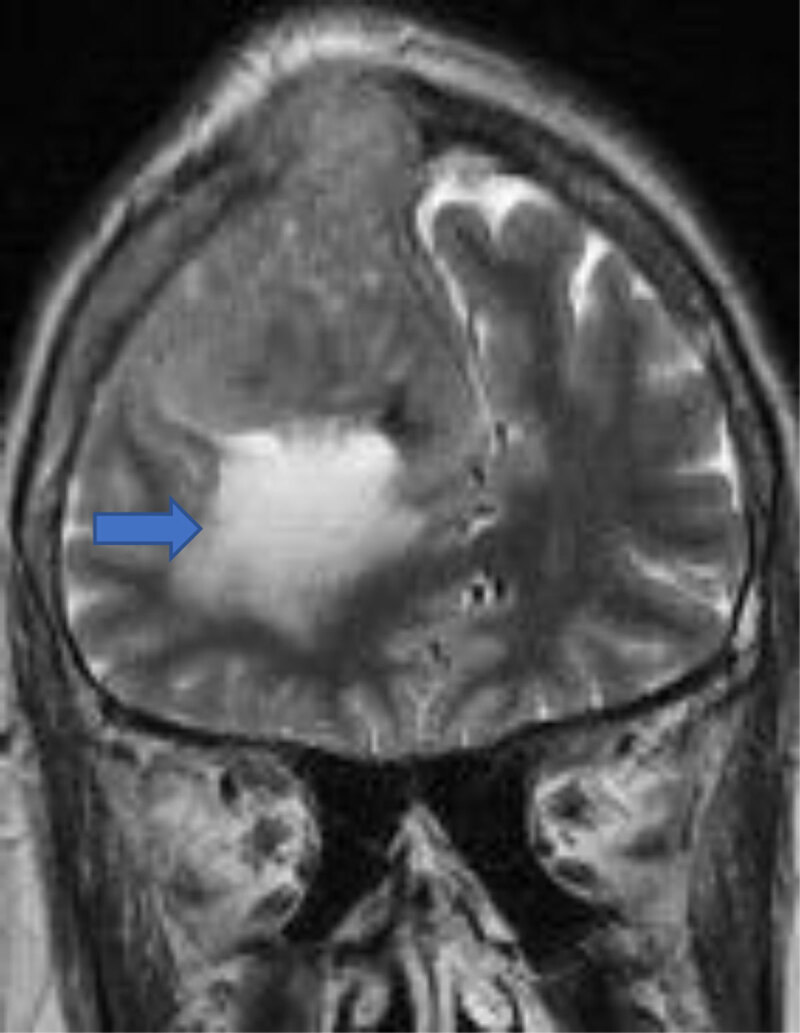
Convexity meningioma with peritumoral edema and (arrow) osseus invasion.

**Peritumoral edema** is seen in almost 50% of lesions. It is related to pial blood supply and vascular endothelial growth factor (VEGF) [1,6]. Infiltrative and microcytic meningiomas are associated with significant edema [7] (***Figure 9***).

**Figure 10:**
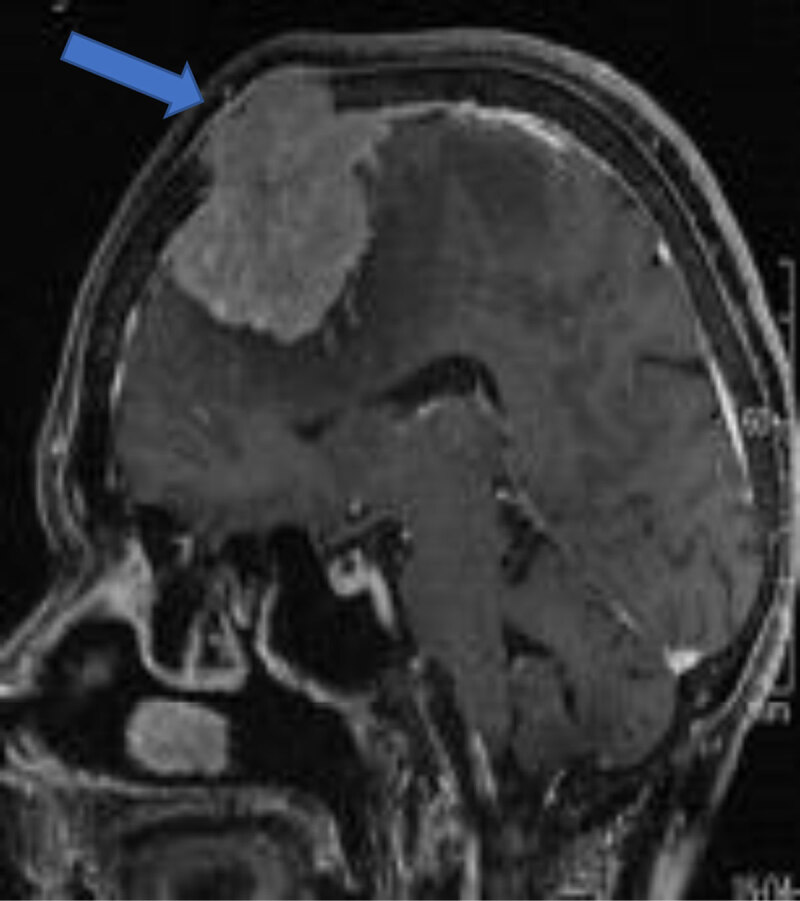
Sagittal post-contrast convexity meningioma with osseous invasion (arrow).

**Bony involvement** occurs with benign and malignant tumors [8] (***Figure 10***).

**Figure 11:**
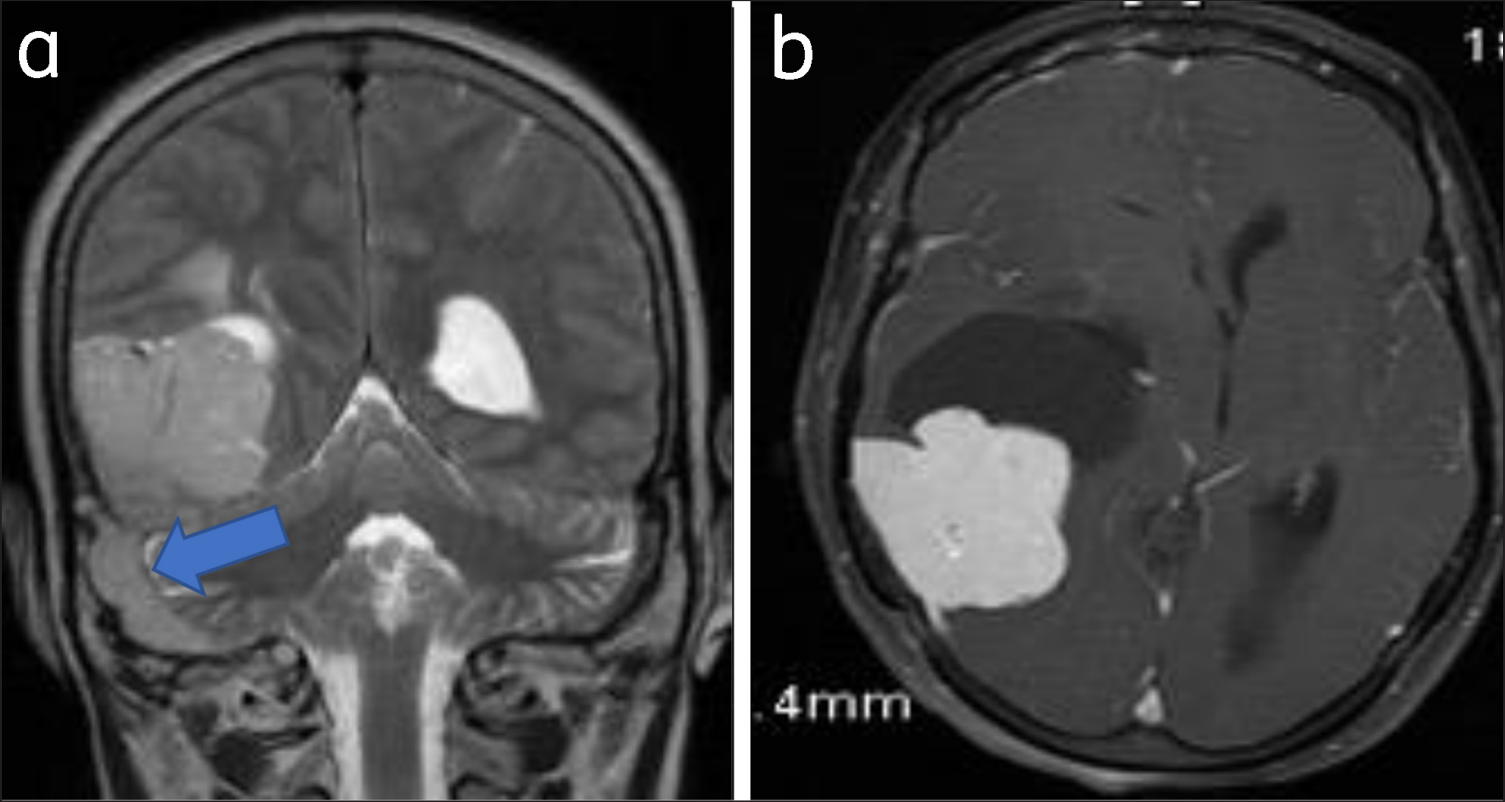
**Meningioma with venous sinus invasion:** Coronal T2WI (**a**) and axial (**b**) post-contrast images show right parieto-occipital meningioma with right sigmoid sinus invasion (arrow).

**Sinus invasion** is the invasion of venous sinuses and is a known complication [2] (***Figures 10, 11***).

**Figure 12:**
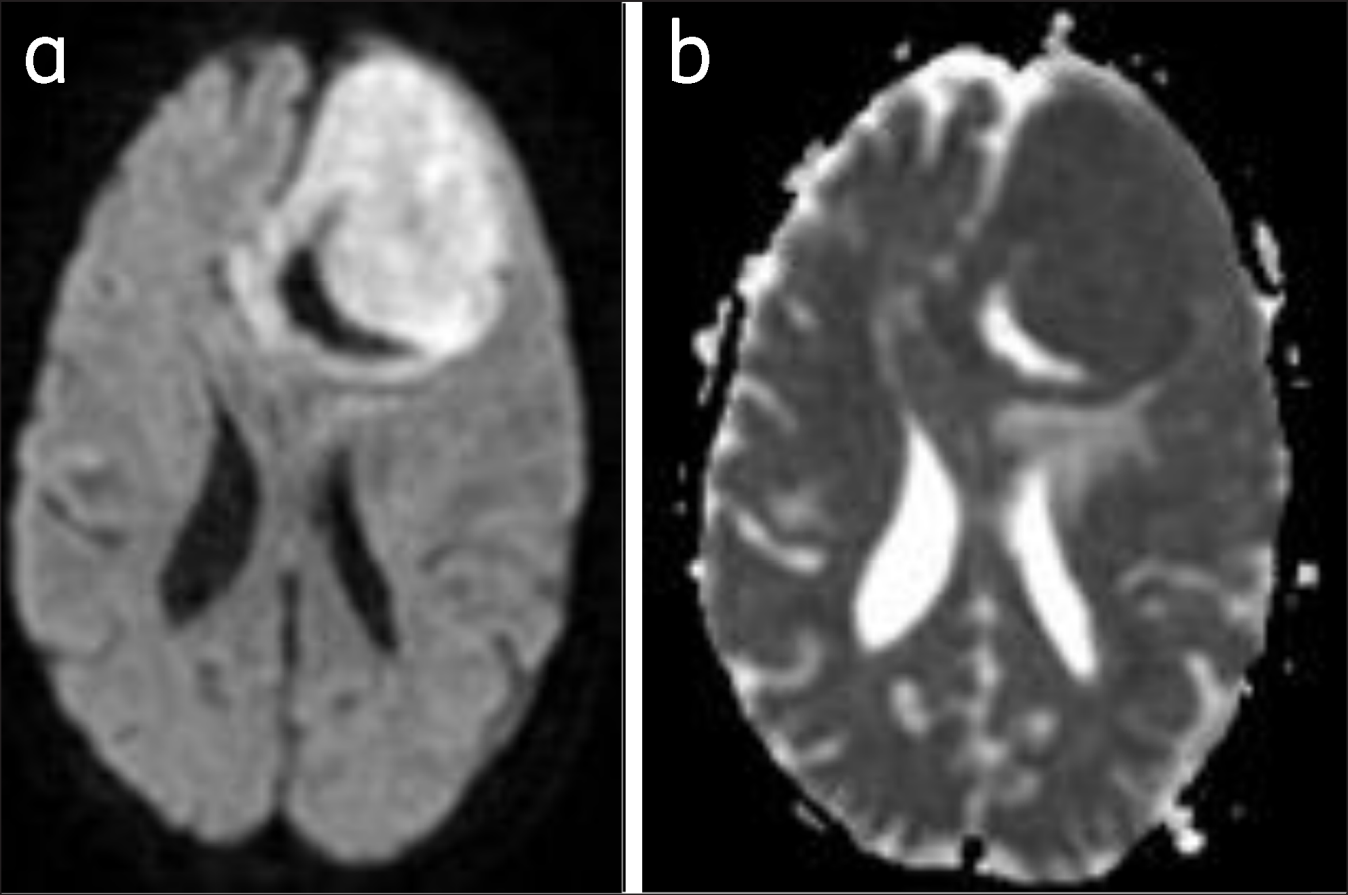
**Meningioma with restriction of diffusion:** Bright on DWI (**a**) and dark on ADC (**b**).

The role of diffusion weighted imaging for grading meningiomas is inconclusive [9] (***Figure 12***).

**Figure 13:**
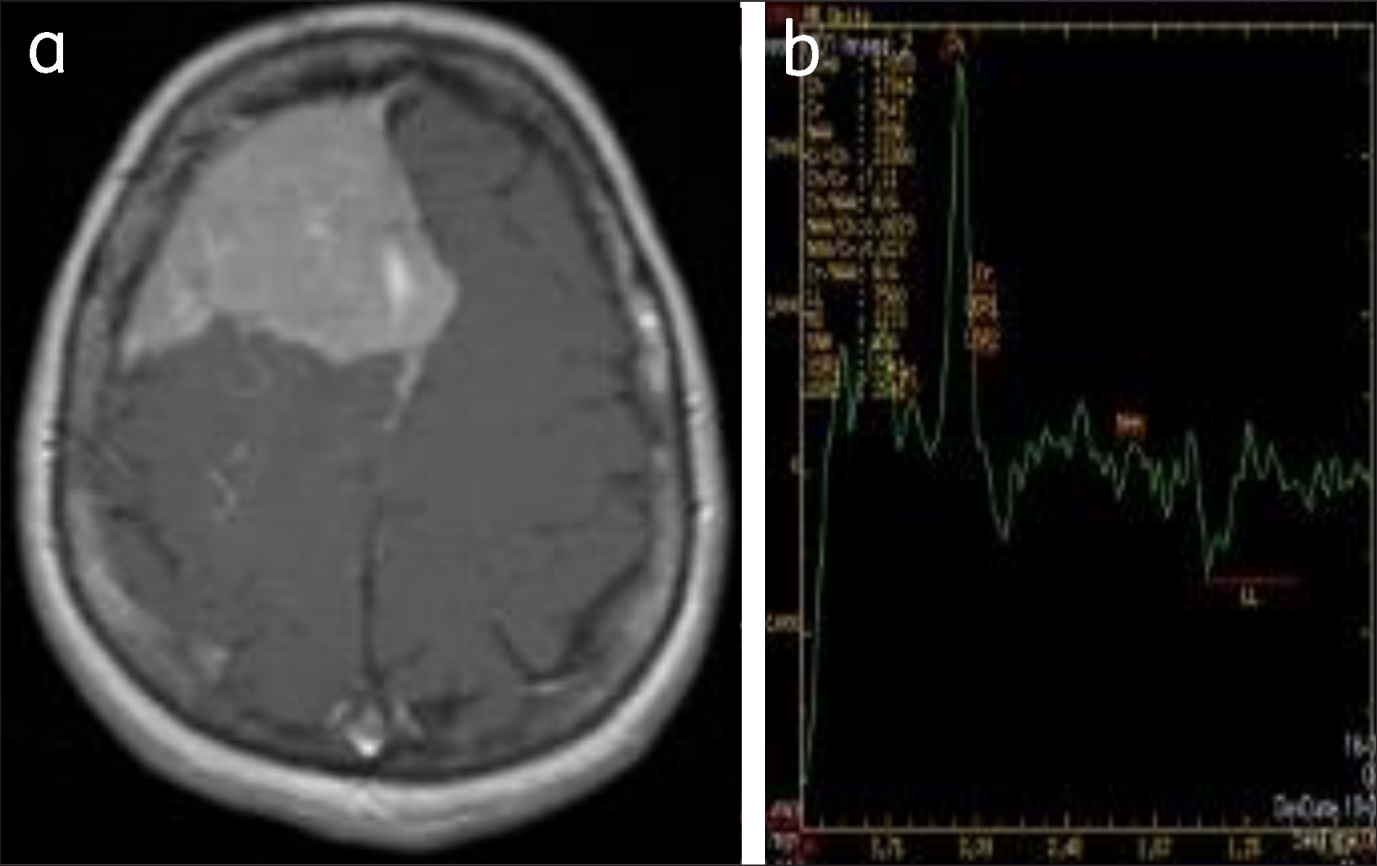
**Convexity meningioma:** (**a**) enhancing right frontal meningioma, (**b**) MRS shows choline peak with reduced NAA.

**MR spectroscopy** shows elevated choline and decreased creatinine in atypical and malignant meningiomas. Alanine is often elevated although glutamate-glutamine and glutathione are more specific [1] (***Figure 13***).

**Figure 14:**
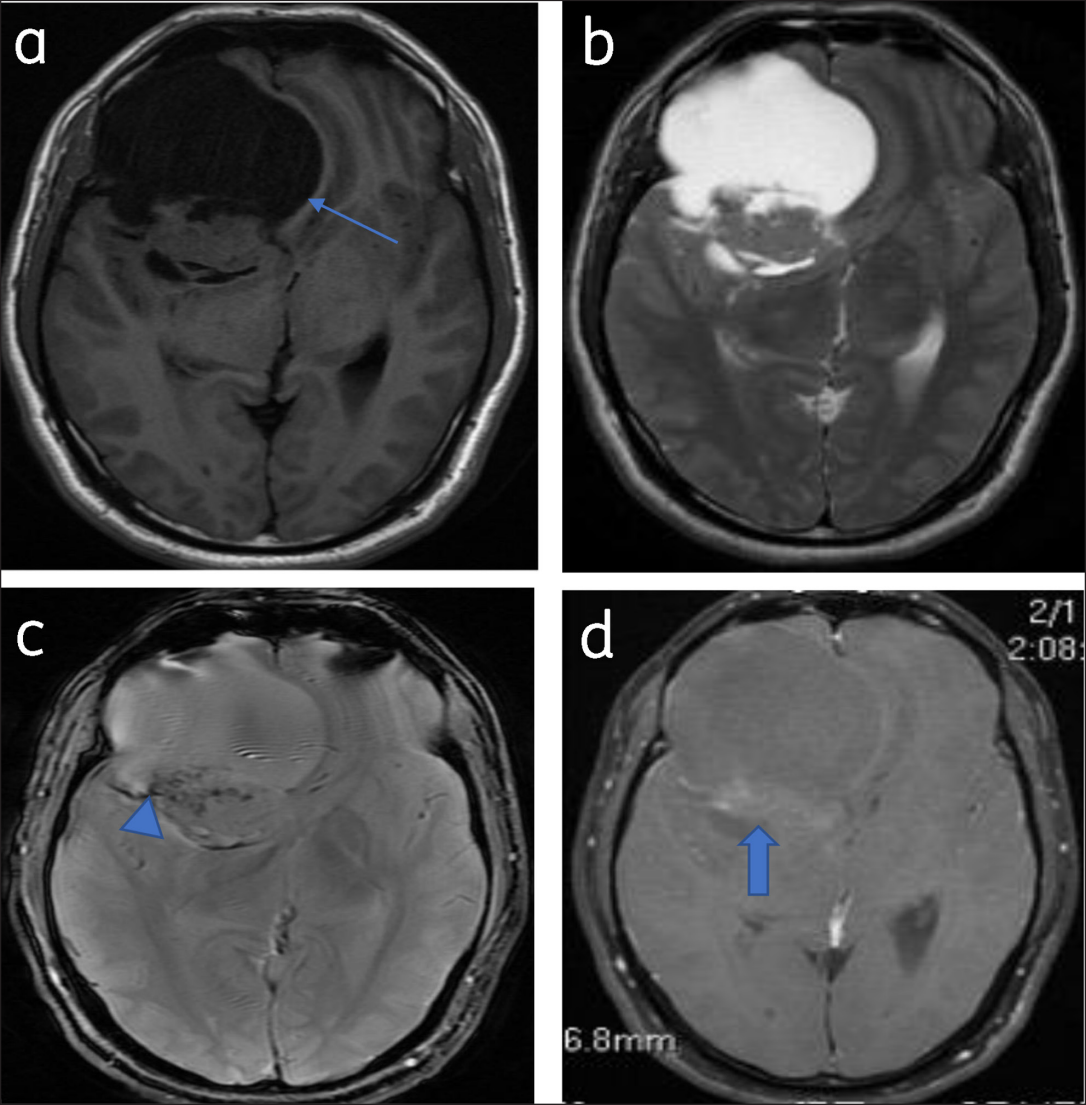
**Predominantly cystic meningioma:** Axial T1WI (**a**) and T2WI (**b**) shows right frontal meningioma with subfalcine herniation (arrow). Blooming (calcification) (arrowhead) seen on T2* image (**c**). Patchy enhancement of solid component (arrow) (**d**).

**Cystic meningiomas** constitute 2 to 4% of intracranial meningiomas. The cystic component may be intra/extratumoral [1] (***Figure 14***).

**Figure 15:**
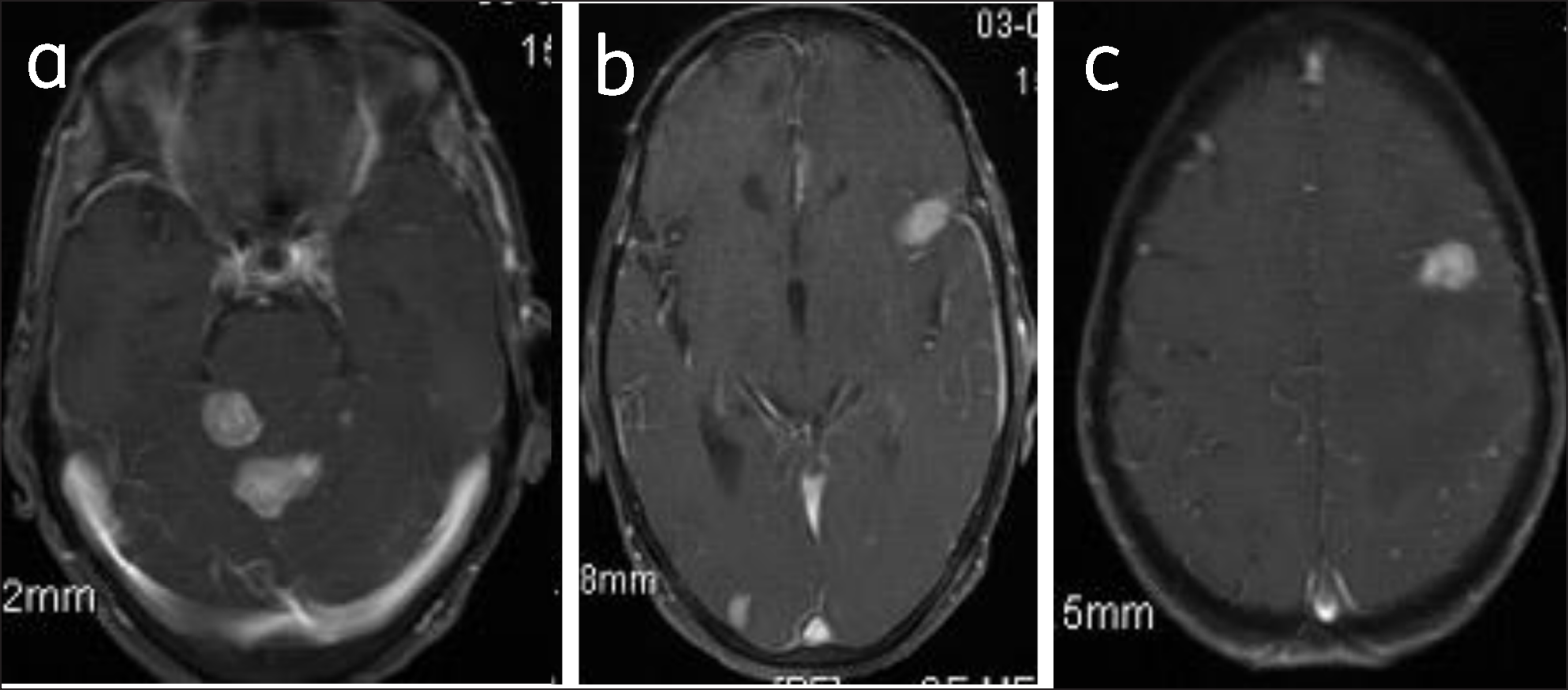
**Meningiomatosis:** Axial post-contrast (**a, b, c**) images show multiple enhancing meningiomas.

**Multiple meningiomas** are seen in association with neurofibromatosis 2 or multiple meningiomatosis syndrome [1] (***Figure 15***).

Intratumoral, subdural, and subarachnoid hemorrhage is an uncommon finding [10]. Lipomatous or lipoblastic meninigioma is a rare subtype [1]. The differential diagnoses include dural metastases, hemangiopericytomas, lymphoma, and neurosarcoidosis [1,2,5].

## Conclusion

Varied appearances can make meningiomas difficult to differentiate from other pathologies.
